# Community-based control of *Aedes aegypti* by adoption of eco-health methods in Chennai City, India

**DOI:** 10.1179/2047773212Y.0000000056

**Published:** 2012-12

**Authors:** Natarajan Arunachalam, Brij Kishore Tyagi, Miriam Samuel, R Krishnamoorthi, R Manavalan, Satish Chandra Tewari, V Ashokkumar, Axel Kroeger, Johannes Sommerfeld, Max Petzold

**Affiliations:** Centre for Research in Medical Entomolgy, Madurai, Tamil Nadu, India

**Keywords:** Dengue, *Aedes aegypti*, Breeding habitats, Community participation, Vector control

## Abstract

**Background:**

Dengue is highly endemic in Chennai city, South India, in spite of continuous vector control efforts. This intervention study was aimed at establishing the efficacy as well as the favouring and limiting factors relating to a community-based environmental intervention package to control the dengue vector *Aedes aegypti*.

**Methods:**

A cluster randomized controlled trial was designed to measure the outcome of a new vector control package and process analysis; different data collection tools were used to determine the performance. Ten randomly selected intervention clusters (neighbourhoods with 100 houses each) were paired with ten control clusters on the basis of ecological/entomological indices and sociological parameters collected during baseline studies. In the intervention clusters, *Aedes* control was carried out using a community-based environmental management approach like provision of water container covers through community actors, clean-up campaigns, and dissemination of dengue information through schoolchildren. The main outcome measure was reduction in pupal indices (pupae per person index), used as a proxy measure of adult vectors, in the intervention clusters compared to the control clusters.

**Results:**

At baseline, almost half the respondents did not know that dengue is serious but preventable, or that it is transmitted by mosquitoes. The stakeholder analysis showed that dengue vector control is carried out by vertically structured programmes of national, state, and local administrative bodies through fogging and larval control with temephos, without any involvement of community-based organizations, and that vector control efforts were conducted in an isolated and irregular way. The most productive container types for *Aedes* pupae were cement tanks, drums, and discarded containers. All ten intervention clusters with a total of 1000 houses and 4639 inhabitants received the intervention while the ten control clusters with a total of 1000 houses and 4439 inhabitants received only the routine government services and some of the information education and communication project materials. The follow-up studies showed that there was a substantial increase in dengue understanding in the intervention group with only minor knowledge changes in the control group. Community involvement and the partnership among stakeholders (particularly women’s self-help groups) worked well. After 10 months of intervention, the pupae per person index was significantly reduced to 0.004 pupae per person from 1.075 (*P* = 0.020) in the intervention clusters compared to control clusters. There were also significant reductions in the Stegomyia indices: the house index was reduced to 4.2%, the container index to 1.05%, and the Breteau index to 4.3 from the baseline values of 19.6, 8.91, and 30.8 in the intervention arm.

**Conclusion:**

A community-based approach together with other stakeholders that promoted interventions to prevent dengue vector breeding led to a substantial reduction in dengue vector density.

## Introduction

An estimated 40% of the global population lives at risk of contracting dengue, which currently is the most important mosquito-borne viral disease and is a serious global public health problem, responsible for 24 000 deaths, 250 000–500 000 hemorrhagic fever cases, and up to 50 million infections annually.^1^ Dengue is transmitted mainly by the vector *Aedes aegypti*, a domestic mosquito species; its breeding sites are closely related to environmental factors linked to human behaviour. Hence, dengue can be conceptualized as an ‘eco-bio-social’ phenomenon.^2^ ‘Eco’-logical factors include climate (rainfall, humidity, temperature, etc.) and the natural and man-made ecological setting. ‘Bio’-logical factors relate to the behaviour of the vector *Ae. aegypti*, and to the transmission dynamics of the disease. Social factors relate to health systems, including vector control and health services and their political contexts (e.g. health sector reforms); to public and private services such as sanitation and sewage, garbage collection, and water supply; and to ‘macro-social’ events such as demographic growth, urbanization, and community and household-based practices, knowledge and attitudes, and how these are shaped by large-scale forces such as poverty, social inequality, and community dynamics. A combined analysis of the eco-bio-social dimensions of dengue revealed the need for community-centred, intersectoral, participatory ecosystem management activities for the control of dengue in Chennai. Hence, this intervention study aims to empower women and student communities to establish a sustainable nature of community-based vector control programme using environmental friendly dengue vector control interventions.

Until a vaccine becomes available for public health use, primary prevention of transmission is crucial to decrease the burden of dengue, and the control of Aedes is the only available strategy.^3^ Elimination of the breeding sites of *Ae*. *aegypti* from the human habitat is the most effective way to manage this vector,^4^,^5^ hence social and behavioural interventions at household level are thought to be the most viable measures for reducing the dengue vector.^6^ Dengue vector control is effective in reducing vector populations, particularly when interventions use a community-based, integrated approach, which is tailored to local eco-epidemiological and sociocultural settings and combined with educational programmes to increase knowledge and understanding of best practice.^7^ Combining top−down control with a sustainable bottom−up programme may represent the optimal approach to dengue vector control^4^ by making the community a partner in the vector control programme. Dengue vector control interventions in various countries include different intervention tools/strategies like the use of insecticide-treated curtains^8^,^9^ and water storage container covers,^9^,^10^ lethal ovitraps,^11^ environmental management approach like cleaning and removal of breeding sites,^3^,^12^ the addition of predators to domestic water containers,^13^–^16^ integrated methods of control,^17^,^18^ intersectoral coordination,^19^ and community empowerment strategy.^20^,^21^ We tested the efficacy of netted frames to cover the key containers combined with a community-centred ecosystem management intervention in reducing dengue entomological indices.

## Materials and Methods

### Study site and its characteristics

**Figure 1:**
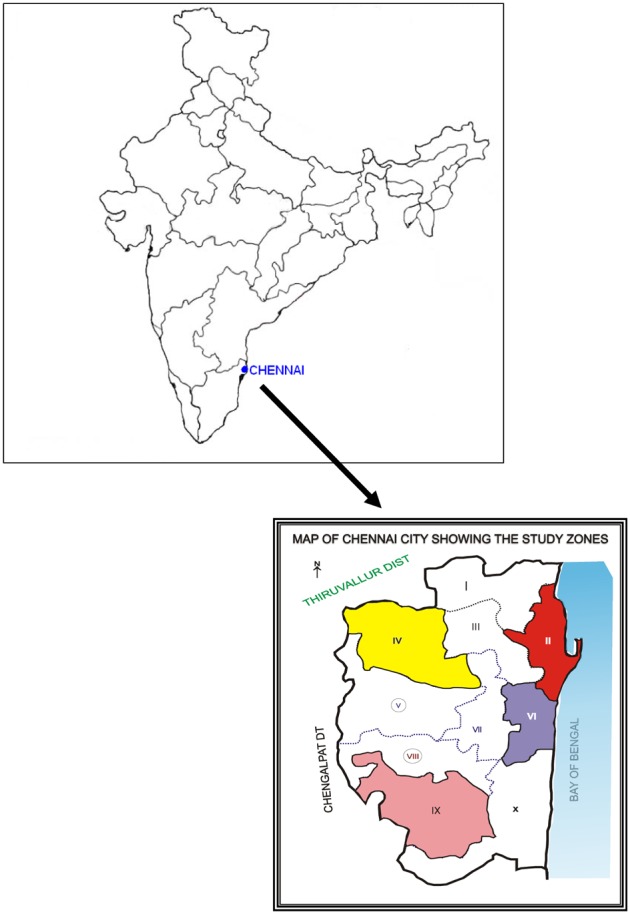
Map of India with study site indicated.

The study was conducted between June 2009 and December 2010 in Chennai city, Tamil Nadu, India. Chennai city is governed by the Corporation of Chennai, which consists of a Mayor and 155 Councillors representing the 155 divisions (all directly elected by the city residents). The Corporation takes care of the civic functions of the metropolis.Chennai city is divided into 10 zones (Fig. 1), and each zone is further subdivided into 12–18 divisions comprising altogether 155 divisions. Each zone has a population of around 0.45 million (0.37–0.58 million) and each division has a population of about 30 000. Tamil Nadu was the most affected of the four southern Indian States, but it was only in Chennai city that high numbers of dengue cases were reported. The city was identified as hyper-endemic as all four dengue serotypes were circulating.^22^,^23^

It rains in Chennai from June onwards, although mainly during the northeast monsoon (September to November). During the study, there was a total of 467 mm rain during the northeast monsoon, and a total of 265 mm during the southwest monsoon (June to August). June was the hottest month and the mean minimum and maximum temperatures were 20.8–28.5 and 29.5–39.1°C respectively, and the relative humidity range was 62–83%.

#### Infrastructure

The city has well-developed urban areas, all with electricity and 90% with paved streets, generally with piped water (72%) and indoor toilets; waste collection was at least once a week in 80% of the study clusters.

#### Housing, socio-economic status

Seventy per cent of the study neighbourhoods (clusters) consisted of residential areas; they were predominantly middle class with good/satisfactory housing — often of two to five storeys and with patios or gardens.

#### Public spaces, tyre capping facilities

Almost half the neighbourhoods had market places (47%), and most of them with schools (80%) and/or small cemeteries (20%). There were relatively few green areas covering 30% of the study sites. Visible garbage dumps and open water pools were found in a third of the study clusters, and tyre capping facilities were found in a quarter of the study clusters.

### Sample size

Calculation of the sample size was related to the post-intervention number of pupae per person using a two-level hierarchical model with clustering at study cluster level, and reflected a desired significance level of 5% and a power of 80%. Based on previous studies, the mean levels of pupae per person in control and intervention areas were assumed to be 3.0 and 0.3, respectively.^2^ For a negative binomial distribution with dispersion coefficient of 0.02 and an intra-cluster coefficient of 0.05, the required number of clusters was 8.9 per study arm when sampling 100 households per cluster; this was increased to 10 clusters per arm. The assumption of a negative binomial distribution is not necessary, but it was intended to add an extra margin to the sample size. The selected clusters and adjacent areas were described in a previous publication.^2^

### Sampling of study neighbourhoods (clusters)

Twenty clusters were randomly selected. A cluster was defined as a neighbourhood of around 100 houses with public (non-residential) areas between or around the houses.

To select the study clusters, a grid with 200 squares was placed on a map of Chennai using Google Earth software, and 20 squares were randomly selected using simple random numbers. The study area was stratified into high and low transmission sites according to reported annual dengue case incidence; half the squares were located in high transmission areas and half in low transmission areas. In each selected square, the lower left hand corner was identified on the map and the exact location determined using GPS methods. This point was then physically located in the city, from where the researcher then identified the nearest point where two streets crossed (one street representing the vertical line of the square on the map and the other the horizontal line). After moving roughly 100 m along the ‘horizontal’ street, the researcher turned left and identified a street running parallel to the first ‘vertical’ street, thus forming a U shape. The researcher then looked for 100 households (houses, flats, and small business units) within the U-shaped area before ‘closing’ the U. Finally, a simple map was drawn for orientation; if the square fell over any open public space (e.g. football ground), the next crossing of two streets was taken. All houses and public and private open spaces were included in the cluster analysis.

### Intervention methods

The stakeholders in the community were identified through consultation meetings to understand the dengue problem in local social systems and to develop strategies to involve them in dengue vector control. The researchers conducted 17 stakeholders consultation meetings with representatives from various groups, namely, community groups, non-governmental voluntary organizations, health staffs, and private garbage cleaning agencies. Various issues on dengue risk in Chennai, vector control activities, garbage cleaning, solid waste management, and prevention of dengue fever were discussed. The stakeholders who were directly or indirectly involved in dengue/mosquito control programmes were identified and mapped. The vector control units in Chennai corporation health department and State public health department are directly involved in vector control. The other stakeholders who have indirect involvement in dengue control are self-help women’s groups, and non-governmental organizations (NGOs) like Madras Christian Council of Social Services, Karunalaya and Arunodhaya, Chennai Corporation school teacher and student community, Neel Metal Fenalica (private agency responsible for garbage cleaning), and Residential Welfare Association.

#### Mobilizing existing women’s self-help groups

Women’s self-help groups (SHGs) are specialist revenue-generating NGOs that help to implement government policies in their areas. In Tamil Nadu, SHGs constitute a strong network under the Tamil Nadu Corporation for Development of Women. Each SHG consists of 12–20 female members and has the objectives of economic, social, and skill development. The members of each SHG meet weekly; they save a minimum amount of money (recurring deposit) each month, and grant credit to any needy member. The nationalized banks provide economic assistance to SHGs, granting loans on easy repayment terms. Members of SHGs are trained and motivated to carry out a number of social and business activities collectively; the women are respected by government organizations and considered responsible for implementing development programmes.

For the dengue intervention programme, the SHG members were briefed by the Tamil Nadu Corporation for Development of Women Project Officer about the objectives, methodologies and vector control strategies which were to be implemented, and the project team established a close rapport with the SHGs. In each selected study cluster, one SHG was identified and a total of ten SHGs were enrolled. One person from each SHG was identified as the dengue focal point to mobilize other members of the group.

#### Involving the community directly

The community participated in the distribution of water container covers and health education materials, and helped the researchers to organize meetings, providing tea and snacks to those who attended the meetings. The women were also actively involved during clean-up campaigns, and took responsibility for cleaning the surroundings. When there were complaints about inadequate removal of solid waste, the researchers helped at the beginning of the project in three occasions the community to get the services.

#### Mobilizing schoolchildren regarding dengue prevention

The project team had repeated interaction regarding the dengue control programme with officials from the Education Department in the Chennai Corporation area. The heads of schools in the intervention clusters (usually one school per cluster) were informed about the objectives of the dengue project, and, in turn, they encouraged the teachers and students (middle level) to participate, especially in the dissemination of dengue-related health messages and environmental sanitation.

#### Distributing information education and communication (IEC) materials in the community

IEC materials that were locally relevant, culturally understandable and acceptable were designed. These included leaflets on household-based dengue transmission, stickers and posters to raise awareness, and wall banners about the vector breeding habitats and prevention options. These materials were developed in conjunction with state health officials engaged in the dengue control programme on the basis of earlier community-based dengue control experiences. The IEC materials were distributed following a well-defined mechanism involving SHGs and schoolchildren in the intervention clusters.

#### Implementing the vector control package: development and provision of water container covers through women’s groups and clean-up campaigns

**Figure 2:**
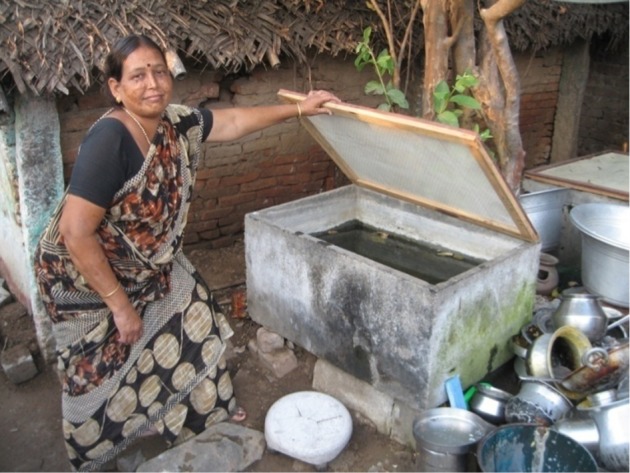
Wooden cover to prevent *Aedes* breeding in cement tanks.

A mechanical vector control tool was designed for cement tanks, which had been shown to be the key productive container. Netted frames of three sizes (small, medium, and large, see Fig. 2) were made locally by sub-contractors and the cost was USD 8 per cover. The local sub-contractors visited each household together with an SHG member to fix the water container cover with metal rings. Additionally the SHG members recommended the disposal of small containers in response to the findings of the baseline survey that plastic/metal drums and grinding stones also belonged to the most productive container types. Small containers had in fact gained greater importance after the Corporation (public sector) had treated the cement tanks with temephos in response to the perceived threat of dengue outbreaks. The cement tanks were the most productive containers (39.9% of pupae) at baseline but now contributed only 7.3% of pupae.

#### Fostering waste disposal and recycling measures

Waste disposal and recycling was managed through partnering with local neighbourhood associations, the health department, and a private sector recycling company. Weekly clean-up campaigns were organized by the project team in the intervention clusters in order to eliminate small discarded containers.

### Research related to the interventions

#### Household survey on people’s knowledge, attitude, and practices

Two surveys on knowledge, attitudes, and practices were carried out at the same time as larval/pupal surveys, at baseline and 6 months after the intervention. A total of 2000 households were interviewed by trained field workers, who used a pre-structured questionnaire that elicited demographic characteristics as well as knowledge about dengue and its prevention.

#### Focus group discussions (FGDs) with health-care providers and community members

A total of ten FGDs were conducted with different members of the community, particularly with women (housewives), socially and economically disadvantaged people, adolescents, and school students, to explore their knowledge and perception of dengue — the prevention methods, vector’s life cycle and breeding habitat, and water storage practices of the community.

#### Key informant interviews with health-care providers and community leaders

Key informant interviews were conducted using semi-structured question guides. Key informants were selected from the intervention clusters, and consisted of those who were working as corporation health officials, councillors, schoolteachers, social workers or basic health workers, or who were working for NGOs/community-based organizations. A total of 20 key informants were selected.

#### Entomological evaluation

The containers were classified according to type, source of water, volume, location, and the presence of vegetation, larvae control measures, and a proper/suitable lid. Only wet containers were registered in all seasons of the survey. For larvae, the surveyor determined the presence or absence of larvae in each container. For pupae, the surveyor counted all the pupae present in each container. A sample of pupae was taken into the laboratory and left to develop into adults. The adults were then identified as to species and sex.

### Data management and analysis of survey data

All data were double checked by field supervisors before data entry, and, to ensure quality, trained personnel carried out double entry of data. All data files were checked and cleaned by data entry supervisors. EpiData 2.0 (www.epidata.dk) software was used for data entry and management as it has range check, skip check, and data export facilities. Study clusters were used as the units for analysis, including by larval indices (container index, house index, and Breteau index) and pupal indices (pupae counts and pupae per person). To evaluate the difference in reduction in larval and pupal indices from baseline to post-intervention between intervention clusters and control clusters, a ‘difference-of-differences’ approach was used. First the average difference between post-intervention and baseline was calculated for the intervention clusters and control clusters, and then the difference of the two differences was calculated (a negative value indicating a larger reduction, or a smaller increase, in the intervention areas).

### Ethical approval

Ethics approval to conduct this research was granted by the Ethics Committee of the Centre for Research in Medical Entomology, and by the WHO Ethical Review Committee. Respondents in the household surveys, key informants, and stakeholders who participated in focus group discussions were provided with an informed consent form and information on the project in the local (Tamil) language.

## Results

### Characteristics of the study population

In the intervention and control areas, 69 and 63.2% of respondents were women, and 31 and 36.8% were men; 61.8 and 58.2% were employed; 18.4 and 21.9% were retired; 3.7 and 3.5% were housewives; and 16.1 and 16.4% were unemployed.

### Gender roles elicited in FGDs, in-depth interviews, community meetings, and observations

The majority of respondents said that men went out to do their occupational work and were involved in income-generating activities while women were responsible for family work such as cooking food, washing clothes, cleaning houses, and caring for children. One women said that ‘In my home, my husband go to sea for fish catching up to fifteen days, then he came back from fishing, he usually went to liquor shop with his peer group. So almost all the days I would take care of my family and children’. Most of the time women were responsible for collecting water from the pipeline, and for the storage and cleaning of water containers. A male respondent stated that ‘In our house we have bore well facility, so we use bore well water for washing clothes, cleaning containers and houses. But we must collect pipe water (Chennai supplied by metro water department) for cooking and drinking. The pipe water is supplied during daytime only, at the time I go to office. So I how can help my wife?’

The social role of men and women was explored by asking for their involvement in local community organizations. The majority of women respondents indicated that they could not be members in any organization, but some said that they were actively involved in SHG meetings. One woman expressed the view that ‘women have more work in family, so we cannot be involved in public activities and generally women are shy to talk with unknown male members’.

Another women explained the positive influence of the SHG by saying: ‘I am now member in SHG, previously I was afraid to go to the bank for deposit or withdraw the amount, but after joining the group I had no fear to go to the bank’.

Thus the survey indicated that the role of women was related to household activities. However, now women are socially and economically empowered by SHGs they are involved in many kinds of social community-welfare activities. Hence the direct involvement of women in the intervention phase had a positive effect on their self-esteem and enhanced their desire to be involved in community matters.

### Knowledge and practice before and after the intervention

Respondents’ knowledge about dengue and how it is transmitted was limited (Table 1). At baseline, although most respondents had heard of the disease, almost half of them did not know that dengue was a serious but preventable illness and that it was transmitted by mosquitoes; very few knew about the mosquito life cycle and breeding behaviour. The same results were also reflected in the FGDs. At baseline, the majority of participants said that drinking unhygienic (not boiled) water in which the worms persist causes dengue fever. One woman said: ‘The children who drink metro water which contains worms get this dengue fever’. Another woman said: ‘mosquitoes sit on waste and polluted materials and again sit on the old cooked rice. If children eat that rice the dengue fever will occur’. A good number of similar explanations were recorded, such as: ‘if we have a close contact with diseased person (sit and work with patients) by the time his body odour come in contact with us the disease is transmitted’, and ‘the mosquitoes exchange different kinds of blood groups through the bites on human being in this process the disease gets transmitted’. A few people said that they knew nothing about dengue. However, many people did say that ‘Dengue fever is spread or transmitted from man to man through mosquitoes’.

**Table 1:** Knowledge about dengue and dengue prevention in the study population before and after the intervention, Chennai

Per cent of cluster dwellers who:	Baseline (*n* = 2000 households)	Intervention areas (*n* = 1000 households)	Control areas (*n* = 1000 households)	Intervention versus control (*P* value)
had heard about dengue	94.0	98.2	90.1	*t* = 3.950 (*P*<0.05*)
considered dengue serious	63.0	90.6	46.1	*t* = 9.908 (*P*<0.05*)
knew that mosquitoes transmit dengue	55.0	90.7	57.4	*t* = 9.198 (*P*<0.05*)
knew that dengue is preventable	55.0	87.4	43.7	*t* = 6.650 (*P*<0.05*)
knew about mosquito life cycle	29.5	89	29.2	*t* = 17.314 (*P*<0.05*)
knew about vector breeding in clean water	26.1	87.5	28.4	*t* = 15.282 (*P*<0.05*)

**Note:** *Significant.

This picture changed quite dramatically after the intervention, when roughly 90% of respondents in the intervention group knew relevant details about the vector and disease transmission while the control group remained practically unchanged (Table 1). After the educational intervention, most respondents said that dengue is caused by mosquitoes, which breed in clean water stored in houses and surrounding areas. One person said: ‘Last year my daughter-in-law was affected by this fever (dengue). At that time blood bleeds from mouth and nose. This fever sucks the blood. My brother and I donated the blood. At that time many children died due to this fever. But I did not know how it occurs. Now I understood it was dengue.’

### Community acceptance of intervention tools

The wooden cement tank covers were in high demand; the community felt that the covers not only prevented mosquitoes from breeding but also gave some protection against dust particles. The covers were frequently checked for repairs (mesh holes), and minor repairs were carried out by the community. The health education materials posted on walls and doors in the intervention houses were periodically checked and all were found to be in good condition; people considered them valuable and said that they would not destroy them.

### Opinions and practices of medical officers (key informant interviews)

All the medical officers concentrated more on malaria and other health programmes related to slum dwellers. They were familiar with the causes of dengue and knew about the *Aedes* mosquito. Most respondents said that treatment for dengue fever was available in government and private hospitals. Health officials felt that, in order to have effective community participation, the community should be educated about the domestic nature of *Aedes*. They also said they had received dengue control guidelines from the National Vector Borne Disease Control Programme in Delhi, and that they tried to apply the recommendations but did not work with other partners or the community.

After the intervention had begun, and with the help of several stakeholders including medical officers, follow-up meetings were held with health officials. The importance of the community-based eco-friendly dengue-control programme was stressed, including how it would lead to reduced use of insecticides, so decreasing environmental pollution.

### Intervention efficacy (reduction in vector density)

Entomological baseline characteristics were similar between the ten intervention and ten control clusters due to the matching process (see methods).

#### Pre-intervention (November–December 2009 rainy season)

Of 4985 *Ae*. *aegypti* pupae collected from 308 positive containers in the intervention areas, 51.2% came from small containers, e.g. flower vases, tyres, discarded containers, and grinding stones. The same pattern was found in control areas: 52.8% of total pupae came from small discarded containers. The productive container types are shown in Table 2. At baseline survey (November–December 2009 wet season), the mean pupae per person index was 1.075.

**Table 2:** Major container types and numbers with pupae found during the wet season base line survey (November–December 2009) in Chennai

			Intervention						Control			
Type of container	Total number of container	Proportion to total containers	Container positive	Proportion to container+ve to total	No. of pupae in positive container	% of all pupae	Total number of containers	Proportion to total containers	Container positive	Proportion of container+ve to total	No. of pupae in positive container	% of all pupae
Cement tank*	73	0.021	19	0.062	366	7.34	58	0.019	22	0.099	431	13.14
Coconut shells	14	0.004	5	0.016	89	1.79	6	0.002	5	0.023	52	1.58
Disused container	156	0.045	87	0.282	1342	26.92	58	0.020	41	0.185	691	21.06
Flower vases	25	0.007	16	0.052	329	6.59	16	0.005	7	0.031	130	3.96
Grinding stone	55	0.016	31	0.101	514	10.31	41	0.014	32	0.144	692	21.09
Plastic/metal drum	260	0.076	63	0.205	1222	24.51	193	0.066	36	0.162	558	17.00
Plastic pot	1434	0.417	17	0.055	205	4.11	1168	0.397	13	0.595	53	1.62
Tyres	24	0.007	21	0.068	279	5.60	14	0.005	13	0.595	166	5.06
Miscellaneous†	1396	0.406	49	0.159	639	12.82	1387	0.472	53	0.239	508	15.48
Total (including all containers	3437		308		4985		2941		222		3281	

**Notes:** *Cement tanks, which were the predominant producers of *Aedes* pupae, had lost their relative importance after the Corporation (public sector) had treated them with temephos, just before our survey in the wet season.

†Miscellaneous containers recorded were ceramic jar, bowl, bucket, metal containers, mud pot, refrigerator trays, and tree holes.

#### Midterm evaluation (after 5 months of intervention)

**Figure 3:**
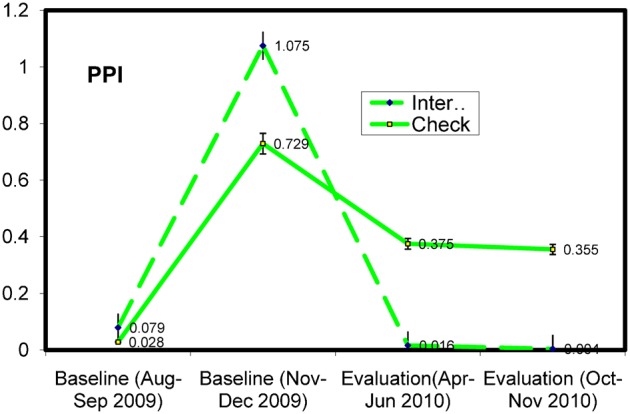
Pupae per person index (95% confidence interval) in intervention and check clusters (August 2009 to November 2010) Chennai, India.

After 5 months of intervention, a midterm entomological evaluation was carried out. Vector infestation levels were significantly lower in the intervention clusters than in the control clusters. The mean pupae per person index declined after 5 months to 0.016 from the highest baseline value of 1.075 (*P*<0.0001) as shown in Fig. 3.

#### Final evaluation (after 10 months of intervention)

After 10 months of intervention, pupae per person index significantly reduced to 0.004 pupae per person from 1.075 (*P* = 0.020) in the intervention clusters (Fig. 3). There were also significant reductions in the Stegomyia indices (house, container, and Breteau indices). The house index reduced to 4.2%, container index reduced to 1.05%, and Breteau index reduced to 4.3 from the baseline values of 19.6, 8.91, and 30.8, respectively in intervention arm (Table 3).

**Table 3:** Summary of entomological indices in the wet season

Indicators	Intervention (1000 houses)		Control (1000 houses)		Difference between arms (intervention and control) wet season	Difference in reduction, baseline to post-intervention, between two arms (*P* value)
	Baseline (November–December 2009)	Evaluation (October–November 2010)	Baseline (November–December 2009)	Evaluation (October–November 2010)		
House index (%)	19.6	4.2	17.2	16.5	−14.7	*P* = 0.012*
Change from baseline		15.4		0.7		
% reduction from baseline		78.6%		4.1%		
						
Container index (%)	8.91	1.05	7.51	5.72	−6.06	*P* = 0.0057*
Change from baseline		7.86		1.79		
% reduction from baseline		88.2%		23.8%		
						
Breteau index	30.8	4.3	22.2	21.4	−25.7	*P* = 0.0011*
Change from baseline		26.5		0.8		
% reduction from baseline		86.0%		3.6		
						
Pupae per person index	1.075	0.004	0.729	0.355	−0.35	*P* = 0.0200*
Change from baseline		1.071		0.374		
% reduction from baseline	99.6	99.6		51.3		

**Note:** *Significant.

## Discussion

Vector control aimed at reducing the density of the dengue vector, *Aedes aegypti*, to low levels is the only currently available measure for preventing dengue transmission. Larviciding, insecticide spraying, and elimination of domestic water containers through community involvement are labour intensive and often difficult to sustain.^9^ The intervention described here was quite feasible to implement as pre-existing structures could be built upon (women’s SHGs, schools, volunteers, and the public sector vector control effort) and existing legislation could be used. The major novelty was to bring different actors together and to use local resources for constructing tailor-made water container covers for rectangular wash basins. The cost of this device (USD 8 per household) could easily be absorbed by the public health sector and the work was carried out by local carpenters.

The main outcomes of the intervention strategy were: reduced vector densities; increased community understanding, acceptance, and support of vector control efforts; continued partnership between the research team, health authorities, and community groups; and community ownership through broader community development issues such as solid waste management with the concepts of ‘reduce, reuse, recycle, recover, and residual management’. It was shown that the intervention strategies, including community involvement through women’s SHGs and creating a dengue partnership with different stakeholders, increased the dynamics of activities against the dengue vectors considerably. The most prominent benefit was the satisfaction created by ‘working together’, which was expressed during FGDs and in-depth interviews. Community mobilization through the work of the women’s SHGs, activities with schools and health authorities, and through distribution of IEC materials in the community, achieved a substantial increase in dengue awareness and understanding in the intervention group compared to the control group. School teachers and students conducted rallies to help with environmental sanitation and dissemination of dengue-related messages.

The provision of water container covers together with clean-up campaigns, resulted in a significant reduction in dengue vector densities. Substantial decrease was seen in pupal indices, particularly the pupae per person index, as a proxy for dengue vector density, in the intervention clusters compared to control clusters. There was demand for the water container covers from the community; users reported a noticeable reduction in larvae and cleaner water in the cement tanks. Using nets to cover water containers is a convenient, inexpensive, and practical way of reducing mosquito populations. A trial study in southern Taiwan^24^ found that covering outdoor buckets with fine nets resulted in significant reduction in density of dengue vectors.

If these methods were adopted by Chennai Corporation along with their routine control programmes, e.g. application of abate in large water storage containers, then the larval breeding would be reduced and the spread of dengue could be contained. In Chennai in 2009 (source: Chennai Corporation), about 16 000 old tyres and 65 tons of waste including broken pots, plastic materials, and coconut shells were disposed of in open spaces; when rainwater stagnates in these articles, they turn into ideal breeding places for mosquitoes. The present study found that after eliminating vector production from large wash basins through use of temephos or — longer lasting — through the use of locally produced wooden lids with a screen, the main production of pupae now comes from tyres, discarded containers, and grinding stones. The study showed a marked and prolonged reduction in the dengue vector population through eco-friendly control methods like solid waste management and clean-up campaigns involving women’s groups and, through them, households.

In conclusion, a community-based approach that promoted interventions to prevent breeding of dengue vectors, and was targeted at multiple stakeholders within communities, led to substantial reduction in the density of dengue vectors. The interventions were developed and implemented based on findings from the baseline study.^2^ Participation of community members was ensured, addressing the fundamental need for people to be involved.
